# Therapeutic effects of mifepristone combined with Gestrinone on patients with endometriosis

**DOI:** 10.12669/pjms.325.10772

**Published:** 2016

**Authors:** Hui-Ling Xue, Ning Yu, Jing Wang, Wan-Jiao Hao, Ye Li, Mei-Yun Liu

**Affiliations:** 1 Hui-Ling Xue, Department of Obstetrics and Gynecology, Affiliated Hospital of Hebei University, Baoding 071000, Hebei Province, P. R. China; 2 Ning Yu, Department of Obstetrics and Gynecology, People’s Hospital of Yi County, Baoding 074200, Hebei Province, P. R. China; 3 Jing Wang, Department of Obstetrics and Gynecology, Affiliated Hospital of Hebei University, Baoding 071000, Hebei Province, P. R. China; 4 Wan-Jiao Hao, Department of Obstetrics and Gynecology, Affiliated Hospital of Hebei University, Baoding 071000, Hebei Province, P. R. China; 5 Ye Li, Department of Obstetrics and Gynecology, Affiliated Hospital of Hebei University, Baoding 071000, Hebei Province, P. R. China; 6 Mei-Yun Liu, Department of Obstetrics and Gynecology, Affiliated Hospital of Hebei University, Baoding 071000, Hebei Province, P. R. China

**Keywords:** Endometriosis, Gestrinone, Mifepristone

## Abstract

**Objective:**

To evaluate the clinical therapeutic effects of mifepristone combined with gestrinone on patients with endometriosis.

**Methods:**

A total of 150 endometriotic patients treated in our hospital between January 2014 and December 2015 were randomly divided into a control group and a treatment group (n=75). The control group began to orally take gestrinone capsules on the second day after menstruation started (2.5 mg/time, twice/week). The treatment group orally took mifepristone tablets (12.5 mg/time, once/day), and the dosage and administration of gestrinone capsules were the same as those of the control group. After 24 weeks of consecutive treatment, the clinical therapeutic effects of the two groups were assessed, and the pelvic symptom score, clinical sign score, serum sex hormone levels and pregnancy outcomes were compared.

**Results:**

The total effective rates of control and treatment groups were 77.3% and 90.7% respectively, between which the difference was statistically significant (P<0.05). After treatment, the scores of pelvic symptoms (dysmenorrhea, dyspareunia, pelvic pain) and clinical signs (pelvic tenderness, induration) significantly reduced (P<0.05). Each score of the treatment group decreased more significantly than that of the control group did (P<0.05). The serum follicle hormone, luteinizing hormone, estrogen and progesterone levels were significantly lower than those before treatment (P<0.05). Each level of the treatment group dropped more significantly than that of the control group did (P<0.05). The pregnancy rates in the 6th and 12th months of follow-up were 28.0% and 13.3% in the control group respectively, and 42.7% and 29.3% in the treatment group respectively. Such rates of the two groups were significantly different at each follow-up time point (P<0.05).

**Conclusion:**

Mifepristone combined with gestrinone had satisfactory clinical therapeutic effects on endometriosis by reducing hormone levels and improving pregnancy outcomes. Therefore, this regimen is worthy of promotion and application in clinical practice.

## INTRODUCTION

Endometriosis refers to the appearance, growth, invasion and repeated bleeding of endometrial tissues (glands and stroma) in parts outside the endometrium, forming nodules and masses that mainly lead to pain and infertility. The clinical symptoms are mainly manifested as progressive dysmenorrhea, irregular menstruation, dyspareunia, infertility and periodic rectal irritation, affecting the health and quality of life of young adult women.^1^,^2^

Endometriosis has become one of the important causes of female infertility, which is mainly treated by combining drugs with surgery, aiming to alleviate clinical symptoms, to relieve pain and to increase the pregnancy rate. Currently, gestrinone and mifepristone are the most frequently used drugs.^3^-^5^ In this study, the clinical therapeutic effects of mifepristone combined with gestrinone on endometriotic patients were evaluated.

## METHODS

### Clinical data

A total of 150 endometriotic patients admitted in our hospital between January 2014 and December 2015 were selected. They were aged between 24 and 40 years, with the mean age of (27.5 ± 5.1 Years). The disease courses ranged from 6 to 72 months, with the mean of (25.3 ± 12.5). Endometrial R-AFS staging: 30 cases in stage I, 28 cases in stage II, 58 cases in stage III and 34 cases in stage IV. All patients met the diagnostic criteria of endometriosis,^6^ and were diagnosed by laparoscopic or three-dimensional ultrasound examination. All patients voluntarily accepted this clinical study, and signed informed consent. The patients during pregnancy or lactation were excluded, and those with drug allergy or contraindications and accompanied by cardiorespiratory, hepatic or renal dysfunction or endocrine diseases were excluded.

Since 1985 the revised American Fertility Society (R-AFS) classification (renamed later American Society for Reproductive Medicine’s classification--ASRM classification) has been a widely accepted system of categorizing the extent of endometriosis. The scoring system gives a detailed description of the location and the severity of the case and correlates to some extent with the degree of pain caused by endometriosis. Based upon collected data, the classification was not found sensitive in predicting pregnancy according to the stage or after treatment of the disease.

### Drugs

Nemestran (gestrinone) capsules were produced by Patheon (UK, 2.5 mg/capsule, batch No. H20080256). Mifepristone tablets were produced by Beijing Zizhu Pharmaceutical Co., Ltd. (25 mg/tablet, batch No. H10950003).

### Grouping and treatment methods

All patients were randomly divided into a control group and a treatment group (n= 75). There were no statistically significant differences between the general information such as age, disease course and endometrial R-AFS stage of the two groups.

The control group began taking gestrinone capsules orally on the second day after menstruation started (2.5 mg/time, twice/week). The treatment group took mifepristone tablets (12.5 mg/time, once/day), and the dosage and administration of gestrinone capsules were the same as those of the control group. Both groups were treated for 24 consecutive weeks.

### Criteria for determining clinical outcomes^7^

Markedly effective: The clinical symptoms and signs all disappear, and the pelvic mass basically disappears; effective: the clinical symptoms and signs are abated, and the pelvic mass is shrunk by over 1/2; ineffective: the clinical symptoms and signs do not change or may be aggravated.

Total effective rate = (markedly effective cases + effective cases)/number of total cases.

### Observation indices

The pelvic symptoms (dysmenorrhea, dyspareunia, pelvic pain) before and after treatment and clinical signs (pelvic tenderness, induration) were scored from 0 to 3 points successively according to the degree of severity, i.e. none, mild, moderate and severe. The serum sex hormone levels of the two groups, including follicle stimulating hormone (FSH), luteinizing hormone (LH), estrogen (E_2_) and progesterone (P), were measured by radioimmunoassay. All patients were followed up for 12 months, and the pregnancy rates at 6 and 12 months were calculated.

### Adverse reactions

Adverse reactions, such as nausea, vomiting, headache, vaginal bleeding and hectic fever, were observed during treatment.

### Statistical analysis

Statistical analysis was performed using SPSS18.0 (SPSS, Inc., Chicago, IL). Continuous data were expressed as mean ± SE, and categorical data are expressed as the median and range. Nonparametric tests (Friedman’s test, Wilcoxon’s signed rank test, and Mann-Whitney test), with and without Bonferroni correction, were used to compare immunostaining scores at various time points. Where there were significant differences in the conclusions, these are noted.

## RESULTS

### Clinical therapeutic effects

With 34 markedly effective and 24 effective cases, the total efficacy of the control group was 77.3%. With 44 markedly effective and 24 effective cases, the treatment group had the total efficacy of 90.7%. The difference was significantly different (P<0.05) (Table-I).

**Table-I:** Clinical therapeutic effects.

*Group*	*Case number (n)*	*Markedly effective (case)*	*Effective (case)*	*Ineffective (case)*	*Total effective rate (%)*
Control	75	34	24	17	77.3
Treatment	75	44	24	7	90.7

### Symptom scores

After treatment, the scores of pelvic symptoms (dysmenorrhea, dyspareunia, pelvic pain) and clinical signs (pelvic tenderness, induration) significantly reduced (P<0.05). Meanwhile, each score of the treatment group decreased more significantly than that of the control group did (P<0.05) (Table-II).

**Table-II:** Symptom scores (χ̄±s, n=75).

*Group*	*Observation time*	*Pelvic symptom score*			*Clinical sign score*	

		*Dysmenorrhea*	*Dyspareunia*	*Pelvic pain*	*Pelvic tenderness*	*Induration*
Control	Before treatment	6.55±1.14	6.11±1.21	6.65±1.34	5.46±1.33	6.02±1.25
	After treatment	2.35±1.01*	1.79±0.72*	2.11±0.57*	2.11±0.51*	2.11±0.56*
Treatment	Before treatment	6.75±0.94	6.27±1.03	6.49±1.23	5.77±1.22	6.05±1.33
	After treatment	0.59±0.24*#	0.72±0.24*#	0.71±0.17*#	0.79±0.21*#	0.89±0.51*#

Compared with the same group before treatment:

* P<0.05; compared with the control group after treatment:

# P<0.05.

### Serum sex hormone levels

The serum FSH, LH, E_2_ and P levels were significantly lower than those before treatment (P<0.05). In the meantime, each level of the treatment group dropped more significantly than that of the control group did (P<0.05) (Table-III).

**Table-III:** Serum sex hormone levels (χ̄±s, n=75).

*Group*	*Observation time*	*FSH (U/L)*	*LH (U/L)*	*E_2_(pmol/L)*	*P (nmol/L)*
Control	Before treatment	9.64±2.11	9.12±0.77	285.22±45.55	6.22±1.12
	After treatment	8.04±2.12*	8.17±1.55*	243.51±51.13*	3.02±1.02*
Treatment	Before treatment	9.89±2.11	9.21±1.23	290.17±50.15	6.27±1.11
	After treatment	6.79±1.99*#	7.35±1.79*#	195.57±49.25*#	2.01±0.86*#

Compared with the same group before treatment:

* P<0.05; compared with the control group after treatment:

# P<0.05.

### Pregnancy outcomes

The pregnancy rates in the 6th and 12th months of follow-up were 28.0% and 13.3% in the control group respectively. There were 31 pregnancy cases within one year, so the total pregnancy rate was 41.3%. The treatment group had the pregnancy rates of 42.7% and 29.3% respectively in the 6th and 12th months of follow-up. There were 54 pregnancy cases within one year, so the total pregnancy rate was 72.0%. Such rates of the two groups were significantly different at each follow-up time point (P<0.05) (Table-IV).

**Table-IV:** Pregnancy outcomes.

*Group*	*N/case number*	*Pregnancy rate in the 6th month*		*Pregnancy rate in the 6th ~ 12th months*		*Total pregnancy rate within one year*	

	*Case No.*	*Pregnancy rate/%*	*Pregnancy Case No.*	*Pregnancy rate/%*	*Pregnancy Case No.*	*Pregnancy rate/%*	*Pregnancy*
Control	75	21	28.0	10	13.3	31	41.3
Treatment	75	32	42.7*	22	29.3*	54	72.0*

Compared with the control group,

* P<0.05.

### Adverse reactions

During treatment, the control group had 9 cases of nausea ad vomiting, three cases of headache, seven cases of vaginal bleeding and two cases of hectic fever, with the incidence rate of 28.0%. The treatment group had 10 cases of nausea and vomiting, 3 cases of headache, 8 cases of vaginal bleeding and 3 cases of hectic fever, with the incidence rate of 31.8%. There was no statistically significant difference between the incidence rates of adverse reactions.

## DISCUSSION

Endometriosis is a common disease among women of childbearing age. Although the disease is benign, it has malignant manifestations which often bring severe physical and psychological burdens to patients, resulting in decreased quality of life. In recent years, the incidence of endometriosis has been increasing annually, which may be associated with endometrial lesions caused by an increase in abortion.^8^,^9^ Endometriosis is easily complicated with infertility, whose causes and mechanisms are not entirely clear, generally being related with retrograde menstruation. For patients with endometriosis associated with infertility, the treatment aims not only to alleviate the symptoms well, but also to ensure normal fertility.^10^,^11^ Currently, there are mainly drug therapy and surgery for the treatment of endometriotic patients with infertility, of which although surgery can relieve pain, it may lead to postoperative infertility due to surgical trauma and easy relapse. Thus, drug therapy has been commonly used in clinical practice, which can alleviate symptoms and improve pregnancy outcomes.

Endometriosis is a hormone-dependent disease. The growth and invasion of endometriosis are dependent on sex hormones which, however, can only work after binding their corresponding receptors. The level up- and down-regulation of sex hormone receptors are associated with sex hormones and related cytokines. The endometrium, as the target tissue of estrogen and progesterone, has corresponding estrogen receptors (ERs) and progesterone receptor (PRs).^4^ The ERα and ERβ levels of eutopic endometrium of normal women of childbearing age vary with the menstrual cycle, i.e. the level of proliferating phase is significantly higher than that of secretory phase, and the ERα level is remarkably higher than that of ERβ. Nevertheless, the ERα and ERβ levels of both eutopic and ectopic endometria of endometriotic patients do not have the cyclical changes.^12^,^13^ PR-A and PR-B are co-expressed in normal endometrium and change along with the menstrual cycle, which constantly rise in the proliferating phase to peak in its later period, decline in the secretory phase, and only have low expressions in the late secretory phase and the early proliferating phase. The level of PR-A is higher than that of PR-B throughout the menstrual cycle.^14^

Mifepristone, with a strong anti-progestational hormone effect, can inhibit ovarian function, reduce ovulation and induce amenorrhea mainly through competition with progesterone for PRs. Meanwhile, it has a non-competitive anti-estrogen effect which can effectively antagonize estrogen and prevent endometrial hyperplasia to promote endometrial atrophy outside the uterine cavity and to alleviate clinical symptoms. Mifepristone is typified by convenience, safety and no significant adverse reactions.^15^,^16^ Gestrinone, as a common drug, is a synthetic triene hormone with anti-progesterone, moderate anti-estrogen and anti-gonadal effects, which can increase the content of free testosterone, reduce the level of sex hormone-binding globulin, suppress the FSH and LH peak values and decrease the LH mean to reduce estrogen levels. In addition, gestrinone has a direct effect on the endometrium and ectopic endometrial receptors, whose roles of anti-progesterone and anti-estrogen lead to endometrial and ectopic endometrial atrophy to achieve therapeutic effects. Its clinical effects have been confirmed in clinical practice. Combining the two drugs can obviously alleviate the clinical symptoms of patients and improve the clinical efficacy and rate of pregnancy, with a synergistic effect.^17^,^18^

In this study, the pelvic symptom scores and overall scores of the treatment group were significantly lower than those before treatment and of the control group, indicating that mifepristone combined with gestrinone significantly mitigated the clinical symptoms of patients. After treatment with mifepristone combined with gestrinone, the FSH, LH, E2 and P levels were significantly lower than those before treatment and of the control group, and the total effective rate and total pregnancy rate were significantly higher than those of the control group, suggesting that this regimen significantly reduced the levels of serum sexual hormones and also markedly improved the clinical efficacy and pregnancy outcomes.

In similar studies, mifepristone was demonstrated to reduce the thickness of the endometrium and to alleviate symptoms, and mifepristone has a significant effect on laparoscopic minimally invasive combined drug therapy.^3^ The antiestrogenic action of gestrinone, associated with its ability to reduce estradiol production by the ovary, may further inhibit endometrial proliferation in endometriosis and allow effective treatment of endometriosis-related pain.^19^ Our preliminary study herein showed that the combined treatment of vaginal gestrinone with mifepristone was very effective for the treatment of endometriosis in patients.

In summary, mifepristone combined with gestrinone had obvious clinical therapeutic effects on endometriosis by reducing sex hormone levels and improving pregnancy outcomes. This strategy is thus worthy of promotion and application in clinical practice.
